# Global mental health and trauma exposure: the current evidence for the relationship between traumatic experiences and spirit possession

**DOI:** 10.3402/ejpt.v6.29126

**Published:** 2015-11-19

**Authors:** Tobias Hecker, Lars Braitmayer, Marjolein van Duijl

**Affiliations:** 1 Department of Psychology, University of Zurich, Zurich, Switzerland; 2 Department of Psychology, University of Konstanz, Konstanz, Germany; 3 vivo international, Konstanz, Germany; 4 Amulet Consultancy for Cultural Psychiatry and Global Mental Health, Leiden, The Netherlands

**Keywords:** spirit possession, possessive trance disorder, trauma exposure, trauma-related disorders, dissociative disorders, PTSD, mental health gap

## Abstract

**Background:**

We present a literature review on trauma exposure and spirit possession in *low- and middle-income countries* (LMICs). Despite the World Health Organization's objective of culturally appropriate mental health care in the Mental Health Action Plan 2013–2020, and the recommendations of the Inter-Agency Standing Committee to consider local idioms of distress and to collaborate with local resources, this topic still receives very little attention. Pathological spirit possession is commonly defined as involuntary, uncontrollable, and occurring outside of ritual settings. It is often associated with stigmatization, suffering, and dysfunctional behavior. While spirit possession has been discussed as an idiom of distress in anthropological literature, recent quantitative studies have presented support for a strong relationship between traumatic experiences and pathological possession states.

**Objective:**

The aim of this review was to investigate this relationship systematically in LMICs, in view of the debate on how to address the mental health gap in LMICs.

**Methods:**

Twenty-one articles, published in peer-reviewed English-language journals between 1994 and 2013, were identified and analyzed with regard to prevalence of possessive trance disorders, patients’ sociodemographic characteristics, and its relation to traumatic experiences.

**Results:**

The review and analysis of 917 patients with symptoms of possessive trance disorders from 14 LMICs indicated that it is a phenomenon occurring worldwide and with global relevance. This literature review suggests a strong relationship between trauma exposure and spirit possession with high prevalence rates found especially in postwar areas in African countries.

**Conclusions:**

More attention for possessive trance disorders in mental health and psychosocial intervention programs in humanitarian emergency settings as well as in societies in transition in LMICs is needed and justified by the results of this systematic literature review.

Global mental health is an emerging field of knowledge highlighting the gaps in mental health services worldwide (Collins, Insel, Chockalingam, Daar, & Maddox, 2013). More than 80% of the global population lives in *low- and middle-income countries* (LMICs), although these countries possess only less than 20% of the resources needed to treat mental disorders. The consequence is that more than 75% of people with mental health disorders in these countries do not receive any official health care at all. A growing amount of research has highlighted this substantial gap between the burden caused by mental disorders and the resources devoted to prevent and treat them (Collins et al., 2013). To bridge these gaps, there is an urgent need for research and action focused on mental health in LMICs.

The World Health Organization (WHO) Mental Health Gap Action Program (mhGAP) provides a strategy, especially for LMICs, for scaling up services for mental, neurological, and substance use disorders (Mathers, Fat, & Boerma, 2008). The vision of the WHO Mental Health Action Plan 2013–2020 (WHO, 2013) is to provide access to culturally appropriate health and social care for all persons suffering from mental disorders. The WHO's plan also fits with the Inter-Agency Standing Committee's (IASC) recommendations to consider local idioms of distress and to collaborate with local, indigenous, and traditional healing systems (IASC Reference Group for Mental Health and Psychosocial Support in Emergency Settings, 2010). Although experiences of spirit possession occur worldwide in many societies (Bourguignon, 1973), and universally used classification systems for mental disorders include criteria to classify dissociative and possessive trance states (American Psychiatric Association, 2000, 2013; WHO, 1992), specific attention on spirit possession and dissociative and possessive trance disorders is still largely lacking in the mhGAP approach and training program. This is even more surprising as anthropologists have already described various forms of pathological possession as an idiom of distress, and there is a now a growing body of research suggesting a relationship between traumatic experiences and pathological forms of spirit possession (Van Duijl, Nijenhuis, Komproe, Gernaat, & De Jong, 2010).

## Pathological forms of spirit possession

The belief that spiritual forces or entities may have an impact on the well-being and personality of individuals is one that is present in cultures around the globe (Bourguignon, 1973). Spirit possession is commonly defined as an altered state of consciousness that involves experiences of being under the control of a powerful entity, such as a god, a demon, a devil, or a spirit (Boddy, 1994). Frequently, this is accompanied by the feeling that the spirit has replaced a person's identity. Spirit possession occurs in many different contexts and manifestations; it provides social functions and is sometimes considered to be culturally accepted, normal, and desirable (Boddy, 1994). Individuals may attribute their illness, their experiences, or the general circumstances in which they are living to the interference of spirits (Cardena, Van Duijl, Weiner, & Terhune, 2009).

The Diagnostic and Statistical Manual of Mental Disorders, fourth edition (DSM-IV; American Psychiatric Association, 2000), included experimental criteria for pathological forms of trance and possession phenomena as examples of dissociative disorders not otherwise specified (DDNOS). *Dissociative trance disorder* (DTD) is defined as a marked alteration of consciousness or loss of the usual sense of identity without replacement by an alternate one, accompanied by a narrowing of awareness of immediate surroundings and stereotyped behaviors or movements which are experienced as being beyond one's control. In *possession trance disorder* (PTD), the usual sense of identity is replaced by another identity. This is attributed to the influence of a spirit, power, deity, or person. The stereotyped and culturally determined behaviors or movements are experienced as being controlled by the possession agent, and there is a full or partial amnesia of the event. The DSM-5 states that distinct personality states of dissociative identity disorder (DID) may be explained as an experience of possession in some cultures. DTD remains classified as DDNOS, and PTD generally is subsumed into the category of the DID (Dalenberg et al., 2012; Van Duijl, Kleijn, & De Jong, 2013). Yet, if the criteria of DID are not entirely fulfilled, PTD may also be classified as DDNOS, for example, if there is no amnesia or if the disruption of identity is less than marked. Furthermore, the DSM-5 introduced a dissociative subtype of posttraumatic stress disorder (PTSD), listing depersonalization and derealization among its symptoms. In some cultures, the dissociative subtype of PTSD may also cover possession phenomena (Sar, Alioglu, & Akyüz, 2014). Another possibility to code possession experiences in the DSM-5 is the new category of acute dissociative reaction to stress (duration of less than 1 month). As transient possession phenomena may not be classified as DID or DDNOS (e.g., as for these diagnoses, the symptoms are required to be chronic), they may be classified as acute dissociative reaction. The International Classification of Diseases, tenth edition (ICD-10), includes trance and possession disorders as a separate entity (WHO, 1992). Very similar to the DSM, the ICD-10 defines trance and possession disorder as a state in which there is a temporary loss of the sense of personal identity and full awareness of the surroundings. Only trance and possession states that are involuntary or unwanted and occur outside of religious or culturally accepted situations are included. From a systematic review that analyzed 28 articles and 402 cases of patients with dissociative trance and possession disorders worldwide, During, Elahi, Taieb, Moro, and Baubet (2011) concluded that dissociative trance and possession disorders are widespread conditions that can be understood as global idioms of distress.

## Trauma exposure and spirit possession

The relation between traumatic experiences and dissociative symptoms is well-established in the literature and can be found in studies from many cultures and countries worldwide (Baita, 2006; Gingrich, 2006; Sar et al., 2014). Dissociation has been described as an adaptive defense mechanism that allows individuals to protect themselves from extreme emotions and arousal when they lack the capacity to integrate adverse experiences (Dalenberg et al., 2012; Seligman & Kirmayer, 2008).

Recent studies have not only shown an enhanced risk of trauma-related disorders, such as PTSD, depression, and substance abuse after exposure to war and violence (Betancourt, Speelman, Onyango, & Bolton, 2009; De Jong, Komproe, & Van Ommeren, 2003; De Jong et al., 2001; Odenwald et al., 2009), but also a close relationship between war-related traumatic experiences and the occurrence of pathological spirit possession (Igreja et al., 2010; Neuner et al., 2012; Van Duijl et al., 2010). In a large survey of the psychological well-being of war-affected youth in Northern Uganda, about 5% of the participants reported being possessed by *cen* spirits (Annan, Blattman, & Horton, 2006). *cen* spirits are the most common and harmful spirits in the conflict region in northern Ugandan. They represent the spirits of dead persons, mostly those that have been murdered. *cen* spirits often possess the spirits of their killers in acts of revenge. However, they may also affect the killers’ clans as well as bystanders who happened to witness the killing or touch or pass by the dead body (Neuner et al., 2012). Yet, *cen* possession may also be regarded as entrance to healing: the spirits indicate the problems that need to be solved. Traditional reconciliation approaches can support negotiation between afflicted parties to settle conflicts and to reconcile. Traditional healers (*ajwaka*), can gain control over *cen* spirits and apply their power for spiritual healing. *cen* spirits can be appeased with cleansing rituals that are performed by healers in the community. Former combatants, in particular, reported being possessed by the spirits of those they had killed. Since local communities maintain that these spirits can spread from one affected person to another, the phenomenon of possession has far-reaching psychological and social implications. Neuner et al. (2012) reported that *cen* possession was especially prominent among child soldiers. This experience of *cen* possession was related to extreme levels of traumatic experiences and predicted suicidal ideation as well as psychosocial dysfunctionality. In addition, Igreja et al. (2010) stated that pathological possession is not a rare, uncommon phenomenon found at the fringes of society, but that it affects broad levels of postwar populations. Indeed, Mozambique, a country that experienced almost three decades of war and devastation, exhibits a possession prevalence rate of more than 18% of the population.

## Objectives

The Global Mental Health Action Plan calls for more research to assess mental health needs in LMICs in order to be able to address them appropriately. Despite the occurrence of pathological forms of spirit possession worldwide, this topic still receives little attention in mental health and psychosocial support (MHPSS) interventions. With this literature review, we aim to present the current findings regarding the relation between traumatic experiences and spirit possession in LMIC settings. We hope that the discussion of our findings can contribute to the debate on global mental health and may add to directions for future research in LMICs.

## Methods

### Search strategy and study selection

We searched electronic literature databases (Medline, PubMed, PsychInfo, and PsychIndex) using the following search terms: *spirit possession*, *pathological possession*, *dissociative trance disorder*, or *possession trance disorder*. Only empirical studies conducted in LMICs published in English-language, peer-reviewed journals qualified for inclusion. Gray literature and unpublished reports were not included in this study. Since the DSM-IV recognized the existence of a pathological possession type, introducing DTD and PTD in 1994, we decided to include only articles published from 1994 until present. Two independent reviewers examined each reference list for other relevant studies. Following these search strategies, 79 studies published between 1994 and February 2014 were found. Of these, 25 were excluded due to a lack of primary data, and 3 because the study was not conducted in a LMIC.

**Fig. 1:**
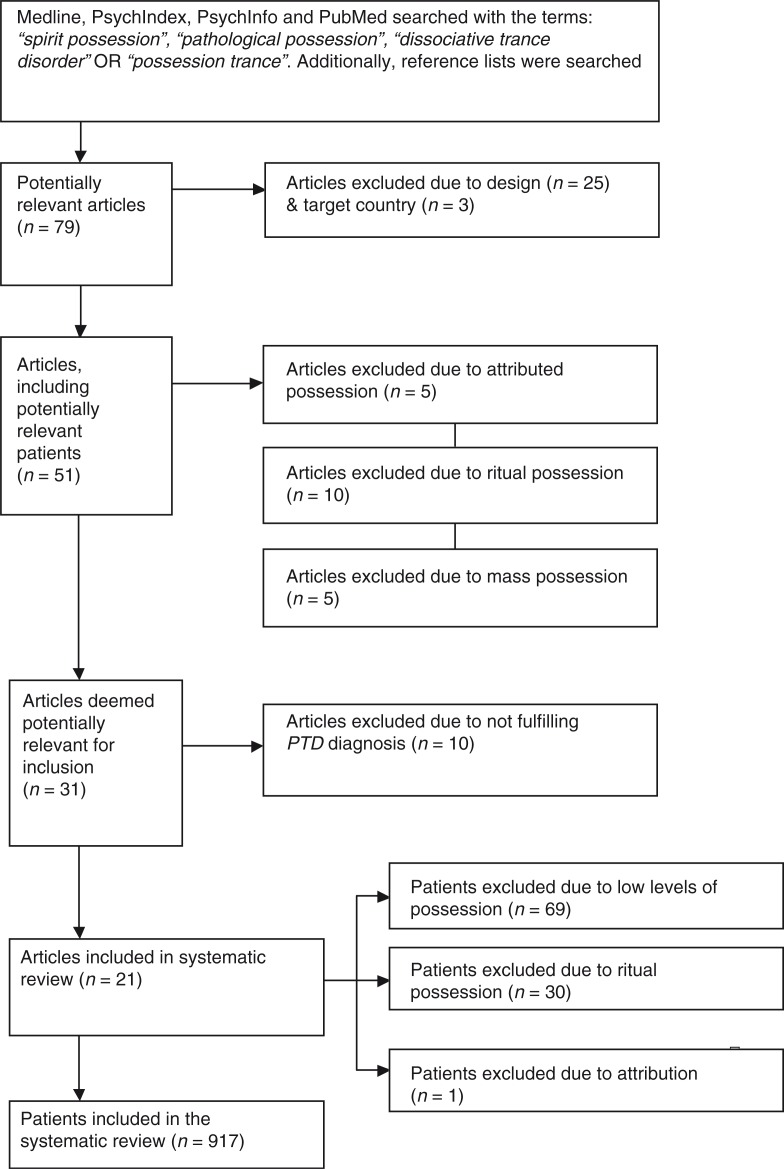
Study selection flowchart.

A study was considered for inclusion if it was in accordance with either the ICD-10 or DSM-IV definition of PTD, reporting symptoms that explicitly refer to the classification of pathological spirit possession such as amnesia, uncontrollable behavior, and replacement of the usual identity by a new identity attributed to a spirit or god. Being considered as pathological implies that the affected individuals described the states of possession as unwanted and troublesome, causing suffering and significant distress or impairment in social or other important areas of functioning. An overview of the selection process is presented in Fig. 1. Table 2 shows an overview of the excluded articles. The application of inclusion and exclusion criteria narrowed the remaining sample down to 21 articles.

A careful analysis of the remaining 21 articles by two independent raters led to the exclusion of certain patients in the included articles. In a sample of 90 persons from Sri Lanka assessing three different groups, all 30 patients of the community sample were excluded since these individuals were known in their communities for their possession states, in which they provided social functions such as oracles or mediumship (Somasundaram, Thivakaran, & Bhugra, 2008). In one study from Uganda, we followed the authors’ approach to distinguish between high and low levels of spirit possession, whereat only the high levels indicated a pathological possession disorder (Neuner et al., 2012). Therefore, 69 sample members were not included in the analysis. In another study, we excluded one of the three reported cases since the description of the patient implied an attributed possession without dissociative symptoms (Pereira, Bhui, & Dein, 1995).

To extract relevant data for the present literature review, two independent raters carefully analyzed all 21 included studies. We extracted data referring to the study characteristics, descriptive statistics (e.g., sex, age, country of residence, identity of possessing agent), potentially traumatizing event types, and the etiological explanations given by the authors. Etiological explanations were categorized as: 1) trauma-related experiences: suggesting a relationship between exposure to traumatic experiences and symptoms of PTD; and 2) cultural conflicts (such as ritual neglect, neglect of responsibilities, land conflicts) and psychosocial stressors (such as familial or marital conflicts and economic or social change): suggesting a relationship between cultural conflicts and/or psychosocial stressors and symptoms of PTD. This latter category includes studies that examine communication theory, which suggests that the possession phenomenon serves as a way for the oppressed and marginalized to express their inner difficulties and problems (see Table 1).

**Table 1:** Overview of the included articles.

Author	Sample	*N*	n inc.	Prev.	Male	Female	Age	Entity	Instrument	Explanation
Bakhshani et al., 2013	Community Rural Iran	4,129	21	0.5% 1.03% (f)	0	21	15–60 Mean=35.76	*(D)jinn* fairy	DES Semi-structured interview	T^a^; PSS^b^
Bayer & Shunaigat, 2002	Clinical Jordan	179	179	—	111	68	9–52 Mean=23.15	*Jinn*	Semi-structured interview	PSS
Castillo, 1994	Case study Rural Sri Lanka India	2	2	—	0	2	15 29	Deceased Demon	—	T
Chand et al., 2000	Clinical Oman	19	19	—	—	—	—	—	—	PSS
Chaturvedi et al., 2010	Clinical India	893	84	9.4%	19	65	—	—	—	PSS
Gaw, Ding, Levine & Gaw, 1998	Rural China	20	20	—	3	17	24–55 Mean=37	Deceased Deity Demon Animal	Structured interview	PSS
Guenedi et al., 2009	Case study Urban Oman	1	1	—	1	0	22	*jinn*	—	Biophysiological Head injury
Hale & Pinninti, 1994	Case study Urban India	1	1	—	1	0	22	Ghost	Interview	
	—									
Igreja et al., 2010	Community Rural Mozambique	941	175	18.6%	—	—	13–60	Ancestral spirits *gamba*	Semi-structured quest. HTQ	T
Khan & Sahni, 2013	Case study Rural Nepal	1	1	—	0	1	20	Deceased	—	High altitude
Khoury, Kaiser, Keys, Brewster, & Kohrt, 2012	Case study Rural Haiti	4	1	0	0	1	20	Evil spirit	Interview	—
Kianpoor & Rhoades, 2006	Rural Iran	10	10	—	1	9	16–32	*jinn*	—	T; PSS
Neuner et al., 2012	Community Uganda	1,113	91	8.2%	22	69	12–25	*cen*	PDS VWAES DHSCL	T
Ng, 2000	Clinical Singapore	55	55	—	43	12	17–69 Mean=28.1	Deities Animal Deceased Evil spirits	Semi-structured interview	PSS
Ng & Chan, 2004	Clinical Singapore	58	58	—	41	17	16–69 Mean=25	Deities Animal Deceased Evil spirits	Semi-structured interview	PSS
Pereira et al., 1995	Case study Rural India Second-generation immigrant	3	2	—	0	2	26 —	Goddess Evil spirit	—	T; PSS
Sar et al., 2014	Community Urban Turkey	628	13	2.1% (f)	0	13	Mean=30.7	*Jinn* Demon Deceased	DDIS SCID CANQ	T
Schieffelin, 1996	Case report Rural Papua New Guinea	4	4	—	2	2	—	Evil spirits	—	PSS
Somasundaram et al., 2008	Clinical Community Rural Sri Lanka	90	60	—	20	40	10–74	Human Ghost Deity	Semi-structured questionnaire	PSS; T
Szabo et al., 2005	Case study Clinical South Africa	1	1	—	0	1	17	—	—	PSS
Van Duijl et al., 2010	Community Rural Uganda	119	119	—	53	66	Mean=38.4	Ancestral spirits, messenger spirits (Emandwa) and halfgods (Bacwezi)	DES SDQ20 HTQ TEC SPQ-Ug CDS-Ug	T; PSS
Total			917		317	406	9–74			

DES, Dissociative Experience Scale; DDIS, Dissociative Disorder Interview Schedule; HTQ, Harvard Traumatic Questionnaire; PDS, Posttraumatic Stress Diagnostic Scale; VWAES, Violence War and Abduction Exposure Scale; DHSCL, Depression Section of the Hopkins Symptom Checklist; DTDIS, Dissociative Trance Disorder Interview Schedule; SCID, Structural Clinic Interview for DSM-IV; CANQ, Childhood Abuse and Neglect Questionnaire; SDQ20, Somatoform Dissociation Questionnaire; SPQ-Ug, Spirit Possession Questionnaire Uganda; TEC, Traumatic Experiences Checklist; CDS-Ug, Checklist Dissociative Symptoms for Uganda; n incl.=subsample included in this systematic review. Explanations given by the authors:

a T, Trauma-Related Explanation;

b PSS, Psychosocial Stressors; Cultural Conflicts; Communication Theory.

## Results

### Study characteristics

The selected studies were published between 1994 and 2013 with the majority after 2003 and differed in study design, sample size, methods, assessment instruments, locations, and research questions (see Table 1). Four studies were case reports (Guenedi et al., 2009; Hale & Pinninti, 1994; Khan & Sahni, 2013; Szabo, Jonsson, & Vorster, 2005) presenting single individuals with spirit possession. Four other articles were case reports presenting 2–10 patients (Brewster et al., 2012; Castillo, 1994; Pereira et al., 1995; Schieffelin, 1996). Four articles were epidemiological studies assessing community-based prevalence rates, two of them in a postwar setting (Igreja et al., 2010; Neuner et al., 2012), the other two in a non-postwar population (Bakhshani, Hosseinbore, & Kianpoor, 2013; Sar et al., 2014). One article used a study design with a control group, matching patients with spirit possession with healthy controls (Van Duijl et al., 2010), whereas another descriptive cross-sectional study compared psychiatric patients, general hospital patients, and community members known for their non-pathological possession states (Somasundaram et al., 2008). Seven articles referred to clinical samples. One of them was a retrospective analysis, assessing the proportions of the different dissociative categories among the registered cases with dissociative disorders (Chand et al., 2000). Four were explicitly referring to the registered clinic patients with DTD or PTD using semi-structured interviews to gain insight about predictors and the clinical and sociodemographic characteristics of spirit possession (Bayer & Shunaigat, 2002; Chaturvedi, Desai, & Shaligram, 2010; Ng, 2000; Ng & Chan, 2004). As shown in Table 1, the selected studies used several assessment instruments, whereas 12 studies, including all case studies, did not provide any information about methods of measurement.

### Descriptive statistics

In total, 917 cases of persons showing symptoms of PTD were found. Among these patients, the sex of the patient was specified in all but two papers (Chand et al., 2000; Igreja et al., 2010). Of the remaining 723 individuals, 44% were male and 56% were female. The age range was between 9 and 74 years, and the mean age ranged from 23.15 to 38.40 years in those studies that reported age-related information (see Table 1).

Cases of patients were found in 14 different countries. Three articles referred to cases from India, and two articles to cases from Iran, Oman, Singapore, Sri Lanka, and Uganda. The other articles reported cases from China, Jordan, Nepal, Turkey, South Africa, Haiti, Papua New Guinea, and Mozambique (see Table 1). The majority of the patients (55%), found in 12 articles, were living in rural areas such as small villages or communities. For 43% of the affected individuals, no specific information about living conditions was provided.

In total, 18 of the 21 included articles gave detailed information about the identity of the possessing agents. Very different kinds of spirits were described in the included studies, ranging from malevolent spirits and demons, such as *jinn* spirits, to goddesses and deities from different pantheons such as Buddhist, Taoist, or Hindu, and the Holy Spirit (Table 1). For example, *jinn* spirits have been described as entities that are living but that are often invisible to human beings. Yet, they also sometimes appear as humans, animals, or black shapes. Hence, they can appear in various shapes, protect cultural and familial values but also have the capability to overpower the human brain. This may lead to different manifestations related to mental illness (Muhammad Gadit & Callanan, 2006). In addition to these entities, spirits of deceased relatives or human ancestors were common, for example, spirits of persons who on death were denied the appropriate cultural rituals because of war or varying religious beliefs, that is, *cen* spirits (see above) or *gamba* spirits. *Gamba* spirits are spirits of male soldiers who died during the civil war in Mozambique. Their bodies were not properly buried, and people living in extreme conditions within the war zones were said to have used the corpses to make medicines to protect themselves against war violence. *Gamba* spirits return to the world of the living to fight for justice. They target women with personal and/or family experiences of extreme suffering, and whose relatives were involved in the use of such protective medicines, or were involved in the murder of the soldiers themselves (Igreja, Dias-Lambranca, & Richter, 2008). Rarely, animal spirits, such as a lion, tiger, or snake, were mentioned.

### Relation between pathological spirit possession and trauma exposure

Nine authors described a relationship between possessive trance states and reported traumatic experiences. This covers 493 cases of pathological spiritual possession, more than half (54%) of the reviewed cases. Exposure to traumatic experiences included war-related experiences, sexual and physical abuse in childhood and in adulthood, the death of relatives, and the murder of close friends. War-related experiences, such as being forced to kill someone or being seriously injured, are reported in three large samples (Igreja et al., 2010; Neuner et al., 2012; Van Duijl et al., 2010), indicating that 41% of the individuals with symptoms of PTD have experienced war-related traumata. Two studies referred to sexual and/or physical abuse in childhood (Sar et al., 2014; Somasundaram et al., 2008), whereas sexual abuse and/or physical abuse in adulthood were reported in three studies (Castillo, 1994; Kianpoor & Rhoades, 2006; Pereira et al., 1995). Beside the association with childhood trauma, Sar et al. (2014) have documented a significant relation between spirit possession and traumatic experiences in adulthood. Other studies reported the exposure to various traumatic experiences (e.g., Bakhshani et al., 2013; Castillo, 1994; Gaw et al., 1998), such as experiencing the death of close family members or witnessing the violent death of another person.

Nevertheless, many authors used alternative, but related and overlapping, approaches to explain the occurrence of possession states. Other disease models that were reported frequently by different scholars focused on psychosocial stressors, cultural conflicts, and/or communication theory. Examples for psychosocial stressors or cultural conflicts were interior conflicts about sexual or moral issues, familial or marital conflicts, debates and uncertainties about cultural or religious traditions and customs, military service, and pressure or tension related to economic or social change and challenges. Communication theory suggests that the possession phenomenon serves as a way for the oppressed and the marginalized to express their inner difficulties and problems when the political or cultural situation may not have allowed them to express their discontent directly. Furthermore, one article debated the possibility of neurobiological reasons, related to an accidental brain injury (Guenedi et al., 2009), while another author considered high altitude sickness as an underlying factor (Khan & Sahni, 2013).

## Discussion

The main goal of this systematic review was to present the current evidence regarding the relation between trauma exposure and spirit possession. Application of our search strategy led to the inclusion of 21 articles since 1994, reporting 917 cases of pathological spirit possession that fulfill the criteria for PTD. Patients were found in 14 different LMICs on three different continents, demonstrating that pathological possession is a phenomenon that occurs around the world (During et al., 2011). The affected individuals were living mainly in rural areas. They were within a wide age range (9–74), of both genders, showed various dissociative symptoms, and the possessing agents were usually experienced in accordance with their cultural background.

### Spirit possession as an idiom of distress or a trauma-related disorder

Two of the reviewed articles assessed the prevalence of spirit possession in non-postwar community samples. Prevalence in a cross-sectional study in northern Iran was about 0.5% in the examined population (*n*=4,129), while a rate of 1% was found for women in the same population (Bakhshani et al., 2013). Sar et al. (2014) reported a prevalence of 2% for an exclusively female sample (*n*=628) from a town in central eastern Turkey. Both rates are smaller than the prevalence rates found in postwar community samples in Mozambique (18%; Igreja et al., 2010) and Uganda (8%; Neuner et al., 2012). Aside from cultural and geographical differences, the level of distress in the particular community or population may impact the prevalence of pathological possession, indicating a relationship between severe stressors, trauma exposure, and spirit possession.

#### Gender and trauma

The included studies have a female to male ratio of 1.28:1. This could support current theoretical, anthropological approaches suggesting that women run a higher risk of developing symptoms of spirit possession than men. However, potential selection bias and the focus on certain at-risk groups in some of the included studies call for caution when interpreting these findings. Bakhshani et al. (2013) did not find a single male patient in their Iranian community sample. Ng and Chan (2004), in contrast, reported that 70% of the registered PTD cases in their study were male. These inconsistent results indicate that it may not be gender, *per se*, which leaves women or men with a general predisposition for spirit possession, but rather the underlying cultural and social circumstances, as well as the psychosocial stressors and traumatic experiences which they entail.

#### Reported trauma experiences

More than 50% of the affected individuals reported some form of psychological trauma; nine authors explicitly referred to a trauma-related disease model for the occurrence of PTD. In addition, many other authors included psychosocial stressors and cultural conflicts in their disease model. High prevalence rates in postwar areas indicate a relation between trauma exposure and pathological spirit possession. Spirit possession seems to be a widespread and potentially underestimated phenomenon in some war-affected populations (Neuner et al., 2012). However, subjects who showed symptoms of spirit possession also reported more traumatic event types in relatively peaceful postwar regions (Van Duijl et al., 2010). In their socio-interpersonal perspective of trauma-related disorders, Maercker and Horn (2013) have stressed the importance of social factors, such as social emotions (shame, guilt, and anger) and social support, both on a family and societal level that impact the severity and course of PTSD symptoms. Social and cultural factors also seem to play an important role in the reported disease models and healing rituals related to PTD and other forms of pathological spirit possession. Concordantly, Baines (2010) has shown how organized violence, such as forcing children to join military forces, resulted in a collapse of kinship networks and social trust. When coming home, the returning abductees are confronted with strong community stigma. Stigma has shown to be an important postconflict factor, profoundly influencing further psychosocial adjustment (Betancourt, Agnew-Blais, Gilman, Williams, & Ellis, 2010). Spirit possession can further increase stigmatization (Kohrt & Hruschka, 2010), and has, therefore, shown to be a risk factor for not participating in communal activities (Igreja, Dias-Lambranca, Hershey, Calero, & Richters, 2009). In this context, spirit possession may not only be related to trauma but also further hinders the recovery efforts and reinforces the impairment of other trauma-related disorders. Thus, more attention may be needed for treatment approaches that involve an interpersonal view of trauma-related disorders (Maercker & Horn, 2013). At the same time, dealing with spirit possession through cultural and religious interventions may also strengthen support and restore relationships within the family and community (Van Duijl, Kleijn, & De Jong, 2014).

### Clinical implications and future research

Spirit possession is a common idiom of distress in the majority of societies in the world (Bourguignon, 1973). Pathological spirit possession can be classified per DSM-5 and ICD-10 as a dissociative disorder. This review underscores evidence for the relationship of PTD with traumatic experiences and high prevalence rates of PTD in postwar settings, especially in African countries. Though epidemiological findings alone may not yet be sufficient to change recommendation for treatment approaches, the current evidence justifies more explicit attention for systematic research of possession and trance disorders in LMICs, particularly in postwar settings.

There are several advantages of attention for PTD in MHPSS programs and mhGAP guidelines: 1) a more appropriate diagnosis and classification may reduce the likelihood of inadequate diagnoses (e.g., psychosis) and subsequent erroneous prescription of psychotropic drugs; 2) a more culturally appropriate diagnosis, understandable in the local context, can facilitate exploration of associated worries and traumata; and 3) the appropriate interpretation and understanding of the presenting symptoms can assist with the identification (with assistance of relatives and community members) of helpful resources in the community. This is also necessary as government mental health services are often scarce and limited in the provision of psychotherapeutic services in many LMICs.

The recently published mhGAP Humanitarian Intervention Guide mentions dissociative symptoms in the module on acute stress including medically unexplained paralysis, inability to speak or see, and pseudoseizures (WHO & UNHCR, 2015). Among the management options, it is mentioned that one should ask for the person's own subjective disease model and consider the use of culturally specific interventions. This is a small step ahead compared with the former mhGAP Intervention Guide for mental, neurological, and substance use disorders in non-specialized health settings (WHO, 2010). As this guide is meant for use in different LMICs, it would be useful to include more symptoms associated with DTD and PTD (Van Duijl et al., 2013, 2010). Such an effort would include local expressions of locally occurring spirits and a mention about local disease models (Van Duijl et al., 2014). When patients present with symptoms of PTD, underlying causes on different levels should be explored systematically. This includes physical problems, emotional stressors and traumatic experiences, cultural conflicts (e.g., forced marriage, unpaid dowries, land conflicts), family problems and intergenerational unresolved issues, economic problems, political oppression, spiritual or ritual neglect, and religious conflicts (Odenwald, Van Duijl, & Schmitt, 2007; Van Duijl, 2014).

Depending on the disease model, treatment for dissociative and possessive disorders may vary from medication and individual trauma-focused therapy to working with families and communities, collaborating with traditional healers and religious leaders, or juridical support and political action (Van Duijl, 2014). In some areas, traditional approaches can offer opportunities for negotiation procedures and reconciliation rituals between conflicting parties (Baines, 2007). Despite the current evidence, more research is needed in different areas. This also requires the use of DTD and PTD as diagnostic categories. Epidemiological and mixed methods research, the overlap with trauma-related diagnostic categories, and cultural and religious interventions are examples of a few areas that require further investigation.

### Limitations

This review has some important limitations that should be noted. First, we only included papers in English language, which may have resulted in a selection bias. Second, we included only patients suffering from PTD. However, the borders between pathological and non-pathological possession are sometimes blurred. Third, the decision not to consider case reports of mass possession and epidemic dissociation led to the exclusion of five articles, probably overlooking single individuals who otherwise would have met the inclusion criteria. Further, spirit possession is associated with specific local cultural, religious, political, and economic contexts (Van Duijl, Cardena, & De Jong, 2005). In this review, we have compared different studies in different contexts, including studies with different agents and with different types and expressions of possessions. For example, the high prevalence in African postwar societies may be related to traumatic experiences, war, and conflict. Yet, another explanation could be that dissociation may be an important part of coping with stress in a specific culture. Therefore, the comparison of these very different studies should be interpreted with caution, as category fallacy cannot be ruled out completely. Also, study designs differed remarkably ranging from single case reports to large community-based epidemiological studies. We cannot completely rule out that this heterogeneity may have biased our findings. Furthermore, our sample included participants with a wide age range (9–74). Though differences may be expected between the different age groups, insufficient data and heterogeneity of the included study did not allow distinct conclusions concerning different age groups. Future research may investigate age-related differences more closely. This systematic review focused on spirit possession in LMIC. Therefore, we did not include studies from high-income countries. However, this should—by no means—indicate that spirit possession is limited to certain cultures or to exotic communities. For example, Ross, Schroeder, and Ness (2013) reported experiences of spirit possession in a sample of predominantly Caucasian American inpatients (see Table 2 for other examples).

**Table 2:** Articles excluded from the systematic research

Author (year)	Reason for exclusion
Carroll, 2004	Attribution of spirit possession
Dein et al., 2008	Attribution of spirit possession
Martinez, 1999	Attribution of spirit possession
Pfeifer, 1999	Attribution of spirit possession
Van Ommeren et al.,2001	Attribution of spirit possession
Mattoo et al., 2002	Mass possession
Nakalawa et al., 2010	Mass possession
Pineros et al., 1998	Mass possession
Sethi et al., 2009	Mass possession
Wedel, 2012	Mass possession
De Jong et al., 2010	Ritual or cult
De Jong et al., 2013	Ritual or cult
Halloy, 2012	Ritual or cult
Masquelier, 2011	Ritual or cult
Moreira et al., 2008	Ritual or cult
Perman, 2011	Ritual or cult
Plancke, 2011	Ritual or cult
Seligman, 2005	Ritual or cult
Seligman, 2010	Ritual or cult
Sidky, 2011	Ritual or cult
Gadit et al., 2006	Not fitting criteria
Dein et al., 2013	Not fitting criteria
Gangdev et al., 1996	Not fitting criteria
Gingrich, 2006	Not fitting criteria
Igreja et al., 2006	Not fitting criteria
Khalifa et al., 2005	Not fitting criteria
Lester, 2008	Not fitting criteria
Rosik, 2004	Not fitting criteria
Ross, 2011	Not fitting criteria
Witzum et al., 1996	Not fitting criteria
Ferracuti et al., 1996	Not conducted in LMIC
Ferracuti & Demarco, 2004	Not conducted in LMIC
Ross et al., 2013	Not conducted in LMIC
Bourguignon, 2005	Study design
Bubandt et al., 2009	Study design
Cardena et al., 2009	Study design
Castillo, 1994	Study design
Chiu, 2000	Study design
Cohen et al., 2008	Study design
During et al., 2011	Study design
Halloy et al., 2012	Study design
Halperin, 1996	Study design
Hegemann, 2013	Study design
Hollan, 2000	Study design
Igreja et al., 2008	Study design
Masquelier, 2008	Study design
Odenwald et al., 2006	Study design
Reis, 2013	Study design
Rhodes, 2005a	Study design
Rhodes, 2005b	Study design
Sersch, 2013	Study design
Somer, 2004	Study design
Suprakash et al., 2013	Study design
Swift, 2006	Study design
Van Duijl et al., 2005	Study design
Van Duijl et al., 2012	Study design
Venkatachalam, 2011	Study design

Finally, it is important to note that many of the included studies are limited in their methodological quality. Some studies did not provide any information about measures that were used. This made it impossible to reconstruct how patients were diagnosed.

## Conclusions

This systematic review and analysis of 917 patients with PTD documented since 1994 indicates that pathological spirit possession is a phenomenon that is occurring globally and has relevance for interventions in many LMICs. Reported prevalence rates differed remarkably and depend upon the cultural background and the particular study populations. High rates were found in postwar areas, indicating a relationship between traumatic experiences and pathological spirit possession. In addition, traumatic experiences and severe psychosocial stressors were included in the disease models in many of the reviewed articles. The findings of the present review are consistent with the view that spirit possession phenomena may be a trauma-related psychopathology: It may be a common idiom of distress or a culture-bound interpretation of trauma-related symptoms in many LMICs. Yet, the impact of potentially traumatizing events related to war, poverty, and societal disruption on the occurrence of DTD and PTD in LMICs also needs further research.

It may help to bridge the mental health gap between what is needed and what is available, if diagnostic categories that are used include locally prevalent and recognizable disorders such as pathological spirit possession states, and interventions that are sensitive to local disease models and treatment options. This may require involving local practitioners and traditional healers when developing mental health services (IASC Reference Group for Mental Health and Psychosocial Support in Emergency Settings, 2010; Van Duijl, 2014). Our findings emphasize the need for further attention and research considering cultural, spiritual, and religious aspects in the development of MHPSS interventions in LMICs.
